# Supplementary material for: The Yorkshire Kidney Screening Trial (YKST): protocol for a feasibility study of adding non-contrast abdominal CT scanning to screen for kidney cancer and other abdominal pathology within a trial of community-based CT screening for lung cancer
Source: BMJ Open. 2022 Sep 20;12(9):e063018. doi: 10.1136/bmjopen-2022-063018 (PMC9490622; doi:10.1136/bmjopen-2022-063018)
Supplement: Author's manuscript [file bmjopen-2022-063018.draft_revisions.pdf]

BMJ Open is committed to open peer review. As part of this commitment we make the peer review history of every article we publish publicly available.

When an article is published we post the peer reviewers' comments and the authors' responses online. We also post the versions of the paper that were used during peer review. These are the versions that the peer review comments apply to.

The versions of the paper that follow are the versions that were submitted during the peer review process. They are not the versions of record or the final published versions. They should not be cited or distributed as the published version of this manuscript.

BMJ Open is an open access journal and the full, final, typeset and author-corrected version of record of the manuscript is available on our site with no access controls, subscription charges or pay-per-view fees (http://bmjopen.bmj.com).

If you have any questions on BMJ Open's open peer review process please email info.bmjopen@bmj.com

BMJ Open

The Yorkshire Kidney Screening Trial (YKST): protocol for a feasibility study of adding non-contrast abdominal CT scanning to screen for kidney cancer and other abdominal pathology within a trial of community-based CT screening for lung cancer

Journal:	*BMJ Open*
Manuscript ID	bmjopen-2022-063018
Article Type:	Protocol
Date Submitted by the Author:	17-Mar-2022
Complete List of Authors:	Usher-Smith, Juliet; University of Cambridge, Department of Public Health and Primary Care<br>Pushpa-Rajah, Angela; University of Cambridge, Department of Surgery<br>Burge, Sarah; University of Cambridge, Cancer Research UK Urological Malignancies Programme, Department of Oncology<br>Burbidge, Simon; Leeds Teaching Hospitals NHS Trust<br>Cartledge, Jon; Saint James's University Hospital<br>Crosbie, Philip; The University of Manchester, Division of Infection, Immunity and Respiratory Medicine<br>Eckert, Claire; University of Leeds<br>Farquhar, Fiona; Leeds Teaching Hospitals NHS Trust<br>Hammond, David; Leeds Teaching Hospitals NHS Trust<br>Hancock, Neil; University of Leeds, School of Food Science and Nutrition<br>Iball, Gareth R; Leeds Teaching Hospitals NHS Trust<br>Kimuli, Michael; Saint James's University Hospital<br>Masson, Golnessa; University of Cambridge, Department of Public Health and Primary Care<br>Neal, Richard; University of Leeds<br>Rogerson, Suzanne; Leeds Teaching Hospitals NHS Trust<br>Rossi, Sabrina; University of Cambridge, Department of Surgery; Cambridge University Hospitals NHS Foundation Trust, Department of Urology<br>Sala, Evis ; University of Cambridge<br>Smith, Andrew; Leeds Teaching Hospitals NHS Trust<br>Sharp, Stephen; University of Cambridge, MRC Epidemiology Unit<br>Simmonds, Irene; University of Leeds<br>Wallace, Tom; Leeds Teaching Hospitals NHS Trust<br>Ward, Matthew; University of Leeds<br>CALLISTER, Matthew; Leeds Centre for Respiratory Medicine, St James's University Hospital<br>Stewart, Grant; University of Cambridge, ;
Keywords:	Kidney tumours < ONCOLOGY, Urological tumours < ONCOLOGY, Computed tomography < RADIOLOGY & IMAGING, Urological tumours < UROLOGY

SCHOLARONE™
Manuscripts

The Yorkshire Kidney Screening Trial (YKST): protocol for a feasibility study of adding non-contrast abdominal CT scanning to screen for kidney cancer and other abdominal pathology within a trial of community-based CT screening for lung cancer

Juliet A Usher-Smith*[1], Angela Pushpa-Rajah*[2], Sarah Burge[3], Simon Burbidge[4], Jon Cartledge[4], Philip AJ Crosbie[5], Claire Eckert[6], Fiona Farquhar[4], David Hammond[4], Neil Hancock[6], Gareth R Iball[4], Michael Kimuli[4], Golnessa Masson[1], Richard D Neal[7], Suzanne Rogerson[4], Sabrina H Rossi[2], Evis Sala[8], Andrew Smith[4], Stephen J Sharp[9], Irene Simmonds[6], Tom Wallace[4], Matthew Ward[6], Matthew E J Callister[4,6], Grant D Stewart[2]

[1]Department of Public Health and Primary Care, University of Cambridge

[2]Department of Surgery, University of Cambridge

[3]Department of Oncology, University of Cambridge

[4]Leeds Teaching Hospitals NHS Trust

[5]Division of Infection, Immunity and Respiratory Medicine, Faculty of Biology, Medicine and Health, University of Manchester, Manchester, UK

[6]Institute of Health Sciences, University of Leeds

[7]College of Medicine and Health, University of Exeter

[8]Department of Radiology, University of Cambridge

[9]MRC Epidemiology Unit, University of Cambridge

*Joint contributors

For peer review only

For peer review only - http://bmjopen.bmj.com/site/about/guidelines.xhtml

Corresponding author: Professor Grant D Stewart, Department of Surgery, University of Cambridge, Cambridge Biomedical Campus, CB2 0QQ, UK. Tel: 01223 769002. Email: gds35@cam.ac.uk

Word count: 3950

For peer review only

For peer review only - http://bmjopen.bmj.com/site/about/guidelines.xhtml

ABSTRACT

Introduction

Kidney cancer (renal cell cancer [RCC]) is the 7th commonest cancer in the UK. As RCC is largely curable if detected at an early stage and most patients have no symptoms, there is international interest in evaluating a screening programme for RCC. The Yorkshire Kidney Screening Trial (YKST) will assess the feasibility of adding non-contrast abdominal CT scanning to screen for RCC and other abdominal pathology within the Yorkshire Lung Screening Trial (YLST), a randomised trial of community-based CT screening for lung cancer.

Methods and analysis

In YLST, ever-smokers aged 55–80 years registered with a general practice in Leeds have been randomised to a Lung Health Check assessment, including a thoracic low-dose CT (LDCT) for those at high risk of lung cancer, or routine care. YLST participants randomised to the Lung Health Check arm who attend for the second round of screening at two years without a history of RCC or abdominal CT scan within the previous six months will be invited to take part in YKST. We anticipate inviting 4,700 participants. Those who consent will have an abdominal CT immediately following their YLST thoracic LDCT. A sub-set of participants and the health care workers involved will be invited to take part in a qualitative interview. Primary objectives are to: quantify the uptake of the abdominal CT, assess the acceptability of the combined screening approach and pilot the majority of procedures for a subsequent randomised controlled trial of RCC screening within lung cancer screening.

Ethics and Dissemination

For peer review only - http://bmjopen.bmj.com/site/about/guidelines.xhtml

YKST was approved by the North West-Preston Research Ethics Committee (21/NW/0021), and the Health Research Authority on 3/2/2021. Trial results will be disseminated at clinical meetings, in peer-reviewed journals and to policy makers. Findings will be made available to participants via the study website (www.YKST.org).

Trial registration numbers

NCT05005195 and ISRCTN18055040

For peer review only

For peer review only - http://bmjopen.bmj.com/site/about/guidelines.xhtml

Strengths and limitations

- YKST is the first study to investigate whether it is acceptable and feasible to combine lung and kidney cancer screening using non-contrast low-dose CT scanning.
- By nesting YKST within an on-going trial of lung cancer screening and performing the abdominal scan immediately after the lung scan, the additional abdominal screening can be conducted with very little additional cost or inconvenience to participants.
- The participants invited to take part in YKST are those who have already consented to take part in a lung screening trial and are considered at high risk of lung cancer so may not be representative of those invited to future cancer screening.
- A nested sub-study will enable assessment of psychological, social and financial harms, and dissatisfaction with health care.

For peer review only - http://bmjopen.bmj.com/site/about/guidelines.xhtml

INTRODUCTION

Kidney cancer (or renal cell cancer [RCC]) is the 7th most common cancer in the UK, and incidence is increasing[1]. As with other cancers, survival is strongly dependent on stage at diagnosis: five-year survival is 87% in stage I compared with 12% in stage IV[1]. Diagnosing RCC at an early stage is therefore central to improving survival. A particular challenge for the diagnosis of RCC is that 60% of patients are asymptomatic, rising to 87% when considering only stage 1 cancers[2]. As a result, up to a third of patients present with incurable stage IV disease [2] and half of all patients developing the disease die from it.

The fact that RCC incidence is increasing, is largely curable if detected early, and most patients are asymptomatic at the time of diagnosis, has resulted in interest from both the scientific community and patient representatives for the development of an RCC screening programme. In particular, screening and early detection of RCC has been identified as a key research priority in three independent priority setting initiatives over the last five years[3–6]. Despite the increasing incidence, however, extrapolating from studies in the USA or Japan, the prevalence of RCC among middle-aged adults within the general population in the UK is estimated to be 0.21% (95% CI, 0.14–0.28%)[7]. This means that approximately 500 individuals would need to be screened to identify one person with a RCC. Targeting screening towards higher-risk individuals is, therefore, likely to be required[8].

The gold standard test for detecting and investigating renal masses is a contrast-enhanced abdominal computed tomography (CT) scan. It is not feasible to use a contrast-enhanced CT as a stand-alone screening test for RCC in the general population due to the relatively high radiation dose and cost, particularly given the low prevalence of RCC. However, using a low-

For peer review only - http://bmjopen.bmj.com/site/about/guidelines.xhtml

dose non-contrast CT scan and combining that with the thoracic low-dose CT (LDCT) scans currently being investigated for lung cancer screening has been proposed as a way to reduce both the costs and radiation, while also potentially offering additional benefit to participants attending lung cancer screening[8].

Thoracic LDCT screening for lung cancer has been shown to reduce mortality in two randomised controlled trials[9,10] and is recommended in adults aged 50-80 years who have a 20 pack-year smoking history or have quit smoking within the past 15 years in the USA by the US Preventive Services Task Force[11]. Several pilots are currently being conducted within England, with Lung Health Checks in some regions of the country in place since Autumn 2019. In addition, the UK National Screening Committee is currently reviewing the effectiveness and cost-effectiveness of lung cancer screening and is due to report shortly. It is possible, therefore, that a comprehensive lung cancer screening programme will be introduced in the UK in the future for older adults with a history of smoking. As older age and smoking are the two strongest risk factors for RCC[12], this population invited for lung cancer screening are also at higher risk of developing RCC.

The potential benefits of using CT to detect RCC have been seen in one of the randomised control trials of lung cancer screening in the USA[13] in which participants diagnosed with RCC within 12 months of the thoracic scan who had a reported abnormality in the upper abdomen had a significantly shorter median time to diagnosis that those without an abnormality in the upper abdomen. However, the thoracic LDCT used within lung cancer screening only includes the upper pole of the kidneys. Additionally, for any screening programme to be successful, eligible individuals need to take up the offer of screening.

For peer review only - http://bmjopen.bmj.com/site/about/guidelines.xhtml

Previous research has shown that providing combined ‘one stop’ cancer screening programmes is viewed positively by members of the public[14] and a survey of over 1000 individuals found that 95% would be “likely” or “very likely” to take up an abdominal CT for RCC screening if it was offered in addition to lung cancer screening[15]. These studies, however, report only intention, and not actual attendance, as no such screening programme currently exists. There are also no studies piloting the additional logistics required for such a combined screening programme.

The Yorkshire Lung Screening Trial (YLST) is a community based, lung screening programme that has recruited individuals who are current or ex-smokers, 55-80 years of age and at high risk of developing lung cancer as defined by the LLP_{v2} score[16], $PLCO_{M2012}$ [18] score or using the 2014 USPSTF criteria[19]. Participants are being invited back for a second thoracic LDCT after two years. Nested within YLST, the Yorkshire Kidney Screening Trial (YKST) will take advantage of this unique opportunity to assess the feasibility and acceptability of offering an additional non-contrast abdominal CT at the same time as the thoracic LDCT as a combined abdominal and lung cancer screening approach and to estimate other key uncertainties needed to inform randomised controlled trials within future screening programmes.

OBJECTIVES

The primary study objectives are:

1. To quantify the uptake of non-contrast abdominal CT to screen for RCC and other abdominal pathology as part of a combined screening modality with thoracic LDCT within a lung health check;

For peer review only - http://bmjopen.bmj.com/site/about/guidelines.xhtml

2. To assess the acceptability to patients of combined lung and RCC screening by non-contrast CT scanning;
3. To evaluate the logistics and acceptability to healthcare professionals involved in the combined lung and RCC screening pathway; and
4. To pilot the majority of procedures for a subsequent full-scale randomised controlled trial of RCC screening by non-contrast CT scanning within lung cancer screening.

The secondary study objectives are to estimate:

1. The prevalence of renal masses and RCC found on non-contrast CT screening in an appropriate age (55-80y) and risk group (smokers and ex-smokers);
2. The stage distribution of RCC identified through non-contrast CT screening;
3. The prevalence of incidental renal findings on non-contrast CT scanning;
4. The prevalence of non-renal findings on non-contrast CT scanning; and
5. The incidence of RCC in the upper pole of the kidney over sequential non-contrast CT scans.

OUTCOME MEASURES

The primary outcome measures are:

1. The proportion of individuals invited to have an additional abdominal CT while attending a second round of lung cancer screening who take up the offer of the abdominal CT;
2. The acceptability to participants of combined lung and RCC screening by non-contrast CT scanning;

For peer review only - http://bmjopen.bmj.com/site/about/guidelines.xhtml

3. The acceptability to healthcare professionals involved in the combined screening approach; and
4. The additional time required for the combined screening approach.

The secondary outcome measures are:

1. The proportion of participants found to have a renal mass or RCC to provide an estimate of the prevalence of RCC found on non-contrast CT screening in 55-80y smokers and ex-smokers;
2. The stage distribution of RCC identified through non-contrast CT screening;
3. The proportion of participants found to have incidental renal findings on non-contrast CT scanning;
4. The proportion of participants with non-renal findings on non-contrast CT scanning; and
5. The proportion of RCCs found on the upper pole of participants at the second thoracic screening round who did not have them in the baseline round, to estimate the incidence of RCC over sequential non-contrast CT scans.

Data will also be collected on further investigations, procedures and management of findings identified on abdominal CT to estimate the individual and health system burden of incidental findings and on the agreement of radiologists reporting the scans and the abdominal CT scan dose and quality to assess the safety. We will also collect long term (10 year) follow-up data on RCC and other abdominal pathology and apply for CAG approval to obtain data on RCC amongst participants within YLST who were not invited to take part in YKST.

For peer review only - http://bmjopen.bmj.com/site/about/guidelines.xhtml

METHODS AND ANALYSIS

Study design

YKST is a non-randomised feasibility study of adding an abdominal CT scan to the thoracic LDCT offered to participants two years after recruitment into the Yorkshire Lung Screening Trial (YLST)[20].

Participants and Recruitment

Participant recruitment is detailed in Figure 1. Participants will be recruited from those attending the second (T2) round of screening within YLST. Full details of YLST are published elsewhere[20]. In brief, YLST is a two-arm (1:1) implementation study using a single-consent Zelen's randomised controlled design with participants randomised to a Lung Health Check or usual care. Participants randomised to the intervention arm are invited to contact a telephone line for a lung cancer risk assessment. Those at high risk of lung cancer are offered a Lung Health Check appointment at a mobile unit sited in convenient community locations, including LDCT screening for lung cancer. The YLST screening programme includes a baseline visit (T0), where participants undergo baseline measurements of height and weight, spirometry (pre-SARS-CoV-2 pandemic), oxygen saturation and exhaled carbon monoxide alongside a smoking cessation intervention and a thoracic LDCT, and a second visit two years later (T2), where participants are offered a further thoracic LDCT.

For peer review only - http://bmjopen.bmj.com/site/about/guidelines.xhtml

Eligibility for YLST is detailed in Figure 1. All those not diagnosed with lung cancer or any other metastatic cancer following the YLST baseline visit (T0) are invited back for T2 between March 2021 and October 2022. The exclusion criteria for YKST at that point are:

- Abdominal or thoracic CT within the last 6 months
- Unable to have an abdominal or thoracic LDCT
- Previous diagnosis of RCC
- Unable to provide informed consent

Invitation process, consent and baseline data collection

The study processes are shown in Figure 2. Participants who attend the mobile van for their YLST T2 visit will be informed of YKST on the van. They will be invited to view the YLST T2 Patient Information video, followed directly by the YKST information video. The YKST video explains the context of the study and the benefits and harms of the additional abdominal CT, including an estimate that in about 5 out of 1000 eligible people the scan may show evidence of RCC, the risks associated with the radiation dose and overdiagnosis and the potential to cause anxiety and worry. Participants are also provided with a written participant information sheet covering the same information. A YLST consultation will follow, at the end of which participants will be asked if they would like to take part in YKST. Translation services are offered to patients where required. Eligibility will be checked and fully informed written consent obtained. As part of this consent, participants will consent to allowing the YKST research team access to their medical records.

For peer review only - http://bmjopen.bmj.com/site/about/guidelines.xhtml

For peer review only

Participants who consent, as well as those who decline the additional scan, will be invited to take part in a qualitative interview. Participants who consent to being contacted about an interview, will be asked to provide their contact details. A separate participant information sheet and consent form will be sent to them and they will be asked to contact a qualitative researcher to arrange an interview.

After providing informed consent, participants will complete a short YKST baseline questionnaire asking whether they have a diagnosis of diabetes or hypertension, whether they take antihypertensive medication, if they have a family history of kidney or pancreatic cancer and their average weekly alcohol consumption. Sociodemographic data (age, sex, socioeconomic status, educational level) and height, weight and smoking status will be obtained from data collected in YLST.

Participants will then be shown to a separate room on the van to have the YLST thoracic LDCT, followed immediately by the YKST abdominal CT. To ensure that only those participants who have consented to YKST receive the additional abdominal CT, radiographers will only perform the abdomen scan for those participants who have i) signed the YKST consent form, and ii) from whom they have received a YKST LDCT request card.

CT scanning protocol

The scanning protocol for the non-contrast abdominal CT will be based on the protocol used for kidney ureter and bladder (KUB) scans within Leeds Teaching Hospital Trust (LTHT) and will be reviewed and monitored by the LTHT medical physics team to ensure that the lowest possible dose allowing interpretable images is used for the YKST abdominal images. A 64-

For peer review only - http://bmjopen.bmj.com/site/about/guidelines.xhtml

channel (or higher) mobile multidetector CT will be used throughout the study. Participants will lie supine on the CT table with arms above their head and thorax and abdomen in the midline of the scanner. Subject comfort will be optimised and maximal inspiration rehearsed prior to the scan to minimise motion during the CT. Imaging will then be performed during suspended maximal inspiration with the standard scanogram used to localise the start and end positions of the scan. No intravenous contrast material will be administered. The kidneys will be scanned in their entirety in a single craniocaudal acquisition and transaxial images of 1mm thickness will be generated, with further reconstructions as necessary. Radiation exposures will be kept as low as possible whilst maintaining good image quality. The average CT dose index (CTDIvol) and Dose Length Product (DLP) for 70-80kg patients will be monitored to ensure they closely match the current typical values of 5mGy and 110mGycm respectively from LTHT scanners. The x-ray tube current (mAs) settings will be automatically varied by the scanner according to participant body habitus. The CT images will then be transferred from the mobile unit to LTH PACS system within 2 days.

CT scan reporting

A team of LTHT uro-radiology consultants will report the abdominal CT scans. They will receive them through the LTHT (PACS) systems and will report them within two weeks of the date of the scan. Scans and reports will be stored on the LTHT electronic patient record (PPM+). The time taken to access and generate the report for these scans will be collected as part of a process evaluation.

The abdominal CT scans will be classified according to one of the five categories below:

For peer review only - http://bmjopen.bmj.com/site/about/guidelines.xhtml

- YKST1 - Normal
- YKST2 - Benign urological finding
- YKST3 - Indeterminate benign finding (i.e. cholecystitis/pancreatitis)
- YKST4 - Possible malignancy outside renal tract or abdominal aortic aneurysm (AAA)
- YKST5 - Possible renal/urological cancer

Normal scan reports (YKST1) will be reviewed and signed off by a senior clinical nurse specialist. A letter will be sent to the patient and their GP, explaining that their scan was normal and that there are no further actions required.

All scans not reported as normal, as well as any normal LDCT scans with discordant reports after second reads (see quality assurance details below), will be reviewed in a screening review meeting (SRM). SRMs will take place twice weekly and will be attended by a consultant urologist with an interest in renal cancer, a senior clinical nurse specialist and a clinical trials administrator. The administrative team will record the agreed outcome and communicate the results to participants and their GPs according to Table 1. Participants will be able to contact the YKST team via the YKST website (www.ykst.org) and the YKST phone number.

For peer review only - http://bmjopen.bmj.com/site/about/guidelines.xhtml

Table 1: Outcomes from Screening Review Meeting

Outcome	Reason	Action	Communication
Normal	No abnormal findings – No Action required	Discharge	Patients and GPs sent letter communicating result
Benign urological and non-urological findings	Benign findings - No Action required	Discharge	Patients and GPs sent letter communicating benign findings and that no further action is required
Indeterminate benign finding	Indeterminate finding requiring further elective investigations	Referral to appropriate speciality coordinated by Consultant Urologist and their delegates. Further tests requested as appropriate following recommendations by radiologist.	YKST lead nurse telephones patients (except for adrenal referrals where referral team contacts patients immediately after the referral is made).<br><br>Patients then contacted by relevant speciality administrative team scheduling appointment and copy sent to GP.
Possible malignancy outside the renal tract or AAA	Abnormality requiring immediate further investigation for possible abdominal cancer or AAA	Fast Track 2 week wait appropriate speciality coordinated by Consultant Urologist and their delegates.	YKST lead nurse contacts patients explaining findings, need for further investigations or onward referral.<br><br>Patients then contacted by relevant speciality administrative team scheduling appointment and copy sent to GP.
Possible renal/urological cancer	Abnormality requiring immediate further investigation for possible renal or urological cancer	During YKST scan review meeting: Consultant Urologist or delegates request contrast scan, refer patient to urology MDT, and assign them to fast track 2 week wait pathway.	YKST lead nurse telephones patients explaining findings and need for further investigations.<br><br>Patients then contacted by relevant speciality administrative team scheduling appointment and copy sent to GP.

AAA – abdominal aortic aneurysm

For peer review only

For peer review only - http://bmjopen.bmj.com/site/about/guidelines.xhtml

Quality Assurance

Ten percent of all normal scans (YKST 1) will be selected at random, re-reported by a different radiologist, and categorised as YKST 1–5 in a second report. The quality of the scans will be assessed both qualitatively using a Likert score from 1 (poor) to 5 (excellent) and quantitatively by selecting a region of interest (ROI). The Likert scale will be recorded by the radiologists for all scans. The ROI assessment will only be performed on the 10% of scans that are second read and will be reported in an addendum to the scan report.

Qualitative interviews

The interviews will take place over the telephone or video call. The interview schedule will be informed by the Theoretical Framework of Acceptability[21] and explore participants' views on the acceptability of the information provided, the consent process, their thoughts on the combined screening approach, and their reasons for accepting or declining the abdominal scan. The interviews will also explore any psychological harm or anxiety that may be experienced by taking part in this combined screening approach. Health care professionals who are involved in the study will also be invited to take part in an interview to assess the acceptability of the combined screening approach to staff members. All interviews will be recorded and transcribed.

Follow-Up

The medical notes of all participants who had an abnormal finding on the abdominal CT will be reviewed six months after the scan by the study team to identify all investigations, procedures, diagnoses and management. Incidental findings will be divided into serious and non-serious based on whether or not they indicate the possibility of a condition which

For peer review only - http://bmjopen.bmj.com/site/about/guidelines.xhtml

would carry a real prospect of seriously threatening life span, or of having a substantial impact on major body functions or quality of life[22]. The classification of findings will be performed by two clinicians independently based on the clinical information within the patient electronic health records and the list of potentially serious / non-serious incidental findings developed in a previous study for abdominal MRI scans based on consultations with radiologists, review of the literature and the German National Cohort's list of imaging incidental findings[22]. Agreement between the two clinicians will be reported by calculating the percentage of findings for which both clinicians agreed on the initial classification. Any discrepancies will be reviewed and discussed with a third clinician.

Long term follow-up will take place between months 20 and 120, and will include: number of kidney and other upper abdominal cancers detected and pathological types; number and details of non-cancer findings; cancer stage at diagnosis; treatments received; date and cause of death.

Psychosocial and other non-physical harms sub-study

A sub-set of approximately 500 participants who have accepted the abdominal CT will be sent a short questionnaire three months and six months after the scan to evaluate outcomes in relation to psychological, social and financial harms, and dissatisfaction with health care. Questionnaires will be sent by post, with participants having the option to complete the questionnaire online. The questionnaire will include validated measures where possible, including the Psychological Consequences Questionnaire (PCQ)[23], the Short form of the Spielberger State Trait Anxiety Inventory (STAI)[24], the EQ-5D-5L[25], and a single question asking how participants would rate their general health now compared to

For peer review only - http://bmjopen.bmj.com/site/about/guidelines.xhtml

For peer review only

before they were invited to take part in YKST. The financial consequences of having the scan will be measured using five questions from a previous study[22] and satisfaction with healthcare using the abbreviated measure to assess trust in the medical profession[26].

Withdrawal of consent

If participants wish to withdraw from the study no further data will be collected on them, though we will keep all data collected to that point. All patient withdrawals will be recorded.

Safety

Adverse events occurring between the time the participants enter the mobile van for their T2 visit and the time that their final result letter is written to them and they are discharged from the study will be recorded and reported in line with Good Clinical Practice.

Sample size

The maximum sample size is limited to those participants who attend for their YLST T2 visit. Approximately 6500 participants were recruited into YLST and it is estimated that 80% of those will attend for the T2 visit. Recruitment began two months into T2 on 10 May 2021 and will run until 31 October 2022. Approximately 4,700 individuals will therefore be eligible for inclusion into YKST. If 80-90% of those take up the additional screening, it will be possible to measure the proportion taking up the additional scan, the primary quantitative outcome of this study, to within 1%. For the qualitative sub-study, the principles of information power[27] will be used to decide when to cease data collection but we anticipate interviewing up to 40 participants. We will purposefully sample participants with the aim to include approximately 20 who accept the additional scan and 20 who do not, with a range of ages, sex, ethnicity and socioeconomic status. For the qualitative interviews

For peer review only - http://bmjopen.bmj.com/site/about/guidelines.xhtml

with healthcare professionals, there are approximately 10 closely involved in the screening process and all will be approached.

Data analysis

Primary outcomes

We will report the proportion of the population attending the T2 screening round who i) are eligible to take part in YKST; ii) are invited to take part in YKST; iii) consent to the additional scan within YKST; iv) decline taking part in YKST. We will also report these proportions by age, sex, smoking status, ethnicity and socio-economic status, and compare those invited who accept the abdominal CT scan between demographic subgroups. The additional time required at each stage of the combined screening approach will be reported. Qualitative data evaluating the acceptability of the combined screening approach will be analysed using Framework analysis, guided by the Theoretical Framework of Acceptability[21]. Each transcript will be read by at least two members of the study team with other members of the study team reading some of the transcripts and contributing to discussions about the overall findings.

Secondary outcomes

Descriptive summaries of secondary outcomes will be reported. When reporting the prevalence and stage distribution of RCC, we will present data among the participants who had the abdominal CT as well as among those from the baseline round of scanning in YLST who either had a renal mass identified in that baseline (T0) lung LDCT or staging investigations for any lung lesions identified and so would have had their full kidneys imaged.

For peer review only - http://bmjopen.bmj.com/site/about/guidelines.xhtml

Patient and Public involvement

Two members of the public were involved in the design of this study and contributed to the research proposal prior to submission for funding. They have also commented on all participant facing documentation and continue to contribute to the study as members of the Independent Trial Steering Committee.

ETHICS AND DISSEMINATION

This study was granted approval by the North West - Preston Research Ethics Committee (reference 21/NW/0021), and the Health Research Authority on 3rd February 2021. It has been adopted onto the National Institute for Health Research trial portfolio (reference 290336). The University of Leeds is the sponsor and together with LTHT acts as joint data controller. The study has been registered on the International Standard Randomised Controlled Trial Number (ISRCTN) (reference ISRCTN18055040) and the National Institutes of Health ClinicalTrials.gov database (reference NCT05005195). The trial will have three committees providing oversight: the Trial Management Group (TMG), the Independent Data Monitoring Committee (IDMC) and an Independent Trials Steering Committee (TSC). The TMG will meet on a monthly basis, and will consist of the co-ordinating team based in Cambridge, members of the YKST team based in Leeds as well as the YLST principal investigator, data manager, project manager and lead nurse. The TMG will provide regular monitoring of the trial and provide clinical, scientific as well as practical advice. The IDMC will meet once or twice a year and will monitor patient safety as well as interim data. The TSC will meet once or twice a year and will provide overall oversight for the trial. It will consider reports from the IDMC, TMG as well as other sources, and will make the final

For peer review only - http://bmjopen.bmj.com/site/about/guidelines.xhtml

decision on whether to recommend early closer or further modifications to the funder. The independent members of the IDMC and TSC will include experts in the field of cancer screening, radiology, renal cancer and statistics. The TSC will also include at least one a patient/public representative.

Findings from the study will be reported in open-access papers in peer-reviewed journals and presented at national and international conferences. We will also provide a lay summary of the findings on the study website (www.YKST.org).

DISCUSSION

As the first study of its kind, YKST will assess the feasibility and acceptability of a combined abdominal and lung cancer screening approach and estimate other key uncertainties needed to inform future randomised controlled trials. Nesting YKST within an on-going randomised lung cancer screening trial also provides a unique opportunity to generate the first cohort of participants invited to undergo screening for RCC. Although not large enough on its own to enable an assessment of whether screening for RCC reduces RCC mortality, this cohort will be a valuable foundation for future research.

Acknowledgements

The authors thank the patient and public representations who have contributed to this study, Phil Alsop and Philip Dondi. They also thank the YLST study team and members of the YKST IDMC (Paul Nathan (Chair), Vicky Goh, Damian Hanbury and Akhtar Nasim) and TSC (Peter Sasieni (Chair), Jonathan Mant, David Nicol, Robert Rintoul, Katie Robb and Jo Waller).

For peer review only - http://bmjopen.bmj.com/site/about/guidelines.xhtml

Authors' contributions

All authors contributed to the design and set up of the study. AP, JUS and GDS wrote the first draft of the manuscript. All authors have contributed to, reviewed and approved the final version of the manuscript.

Funding

This study is funded by Yorkshire Cancer Research grant number L403C. The qualitative sub-study is funded by a grant from Kidney Cancer UK. GDS is supported by The Mark Foundation for Cancer Research, the Cancer Research UK Cambridge Centre [C9685/A25177] and NIHR Cambridge Biomedical Research Centre (BRC-1215-20014). PAJC is supported by the Manchester National Institute for Health Research Manchester Biomedical Research Centre (IS-BRC-1215-20007). The views expressed are those of the author(s) and not necessarily those of the NIHR or the Department of Health and Social Care.

Patient consent for publication

Not required

Data sharing

In order to meet our ethical obligation to responsibly share data generated by clinical trials, YKST operates a transparent data sharing request process. Anonymous data will be available for request once the study has published the final proposed analyses. Researchers wishing to use the data will need to complete a Request for Data Sharing form describing a

For peer review only

methodologically sound proposal. The form will need to include the objectives, what data are requested, timelines for use, intellectual property and publication rights, data release definition in the contract and participant informed consent etc.. A Data Sharing Agreement from the Sponsor may also be required.

Competing interests

All authors have completed the Unified Competing Interest form at www.icmje.org/coi_disclosure.pdf (available on request from the corresponding author). GDS has received educational grants from Pfizer, AstraZeneca, and Intuitive Surgical; consultancy fees from Pfizer, Merck, EUSA Pharma, and CMR Surgical; travel expenses from Pfizer; and speaker fees from Pfizer.

All other authors declare that (1) they have no support from or relationships with companies that might have an interest in the submitted work in the previous 3 years; (2) their spouses, partners, or children have no financial relationships that may be relevant to the submitted work; and (3) they have no non-financial interests that may be relevant to the submitted work.

The corresponding author has the right to grant on behalf of all authors and does grant on behalf of all authors, an exclusive licence on a worldwide basis to the BMJ Publishing Group Ltd and its Licensees to permit this article (if accepted) to be published in BMJ editions and any other BMJPGL products and sub-licences to exploit all subsidiary rights, as set out in our licence (http://resources.bmj.com/bmj/authors/checklists-forms/licence-for-publication).

For peer review only - http://bmjopen.bmj.com/site/about/guidelines.xhtml

The corresponding author affirms that the manuscript is an honest, accurate, and transparent account of the study being reported; that no important aspects of the study have been omitted; and that any discrepancies from the study as planned (and, if relevant, registered) have been explained.

REFERENCES

1 Kidney cancer statistics | Cancer Research UK. https://www.cancerresearchuk.org/health-professional/cancer-statistics/statistics-by-cancer-type/kidney-cancer (accessed 30 May 2019).

2 Vasudev NS, Wilson M, Stewart GD, *et al.* Challenges of early renal cancer detection: Symptom patterns and incidental diagnosis rate in a multicentre prospective UK cohort of patients presenting with suspected renal cancer. *BMJ Open* 2020;**10**:1–9. doi:10.1136/bmjopen-2019-035938

3 Rossi SH, Blick C, Handforth C, *et al.* Essential Research Priorities in Renal Cancer: A Modified Delphi Consensus Statement. *Eur Urol Focus* Published Online First: February 2019. doi:10.1016/j.euf.2019.01.014

4 Rossi SH, Fielding A, Blick C, *et al.* Setting Research Priorities in Partnership with Patients to Provide Patient-centred Urological Cancer Care. *Eur Urol* 2019;**75**:891–3. doi:10.1016/j.eururo.2019.03.008

5 Rini B, Abel EJ, Albiges L, *et al.* Summary from the Kidney Cancer Association's Inaugural Think Tank: Coalition for a Cure. *Clin Genitourin Cancer* 2021;**19**:167–75. doi:10.1016/J.CLGC.2020.10.005

6 Jones J, Bhatt J, Avery J, *et al.* The kidney cancer research priority-setting partnership: Identifying the top 10 research priorities as defined by patients, caregivers, and

For peer review only - http://bmjopen.bmj.com/site/about/guidelines.xhtml

expert clinicians. *Can Urol Assoc J* 2017;**11**:379–87. doi:10.5489/cuaj.4590

7 Rossi SH, Hsu R, Blick C, *et al.* Meta-analysis of the prevalence of renal cancer detected by abdominal ultrasonography. *Br J Surg* 2017;**104**:648–59. doi:10.1002/bjs.10523

8 Usher-Smith J, Simmons R, Rossi S, *et al.* Current evidence on screening for renal cancer. *Nat Rev Urol* Published Online First: 2020. doi:10.1016/B978-0-323-47873-1.00100-5

9 de Koning HJ, van der Aalst CM, de Jong PA, *et al.* Reduced Lung-Cancer Mortality with Volume CT Screening in a Randomized Trial. *N Engl J Med* 2020;**382**:503–13. doi:10.1056/nejmoa1911793

10 The National Lung Screening Trial Research Team. Reduced Lung-Cancer Mortality with Low-Dose Computed Tomographic Screening. *N Engl J Med* 2011;**365**:395–409.

11 Krist AH, Davidson KW, Mangione CM, *et al.* Screening for Lung Cancer: US Preventive Services Task Force Recommendation Statement. *JAMA - J Am Med Assoc* 2021;**325**:962–70. doi:10.1001/jama.2021.1117

12 Rossi SH, Klatte T, Usher-Smith J, *et al.* Epidemiology and screening for renal cancer. *World J Urol* Published Online First: 2018. doi:10.1007/s00345-018-2286-7

13 Pinsky PF, Dunn B, Gierada D, *et al.* Incidental renal tumours on low-dose CT lung cancer screening exams. *J Med Screen* 2017;**24**:104–9. doi:10.1177/0969141316657115

14 Bobridge A, Price K, Gill TK, *et al.* Influencing cancer screening participation rates-providing a combined cancer screening program (a 'One Stop' shop) could be a potential answer. *Front Oncol* 2017;**7**:1–7. doi:10.3389/fonc.2017.00308

15 Harvey-Kelly LLW, Harrison H, Rossi SH, *et al.* Public attitudes towards screening for

For peer review only - http://bmjopen.bmj.com/site/about/guidelines.xhtml

For peer review only

kidney cancer: an online survey. *BMC Urol* 2020;:1–10. doi:10.1186/s12894-020-00724-0

16 Cassidy A, Myles JP, Van Tongeren M, *et al.* The LLP risk model: an individual risk prediction model for lung cancer. *Br J Cancer* 2008;**98**:270. doi:10.1038/SJ.BJC.6604158

17 Marcus MW, Chen Y, Raji OY, *et al.* LLPi: Liverpool Lung Project Risk Prediction Model for Lung Cancer Incidence. *Cancer Prev Res (Phila)* 2015;**8**:570–5. doi:10.1158/1940-6207.CAPR-14-0438

18 Tammemägi MC, Katki HA, Hocking WG, *et al.* Selection Criteria for Lung-Cancer Screening. *N Engl J Med* 2013;**368**:728–36. doi:10.1056/nejmoa1211776

19 Moyer VA. Screening for lung cancer: U.S. preventive services task force recommendation statement. *Ann Intern Med* 2014;**160**:330–8. doi:10.7326/M13-2771

20 Crosbie PA, Gabe R, Simmonds I, *et al.* Yorkshire Lung Screening Trial (YLST): protocol for a randomised controlled trial to evaluate invitation to community-based low-dose CT screening for lung cancer versus usual care in a targeted population at risk. *BMJ Open* 2020;**10**:e037075. doi:10.1136/bmjopen-2020-037075

21 Sekhon M, Cartwright M, Francis JJ. Acceptability of health care interventions: A theoretical framework and proposed research agenda. *Br J Health Psychol* 2018;:519–31. doi:10.1111/bjhp.12295

22 Gibson LM, Littlejohns TJ, Adamska L, *et al.* Impact of detecting potentially serious incidental findings during multi-modal imaging [version 3; referees: 2 approved, 1 approved with reservations]. *Wellcome Open Res* 2018;**2**. doi:10.12688/wellcomeopenres.13181.3

For peer review only - http://bmjopen.bmj.com/site/about/guidelines.xhtml

23 Cockburn J, De Luise T, Hurley S, *et al.* Development and validation of the PCQ: a questionnaire to measure the psychological consequences of screening mammography. *Soc Sci Med* 1992;**34**:1129–34. doi:10.1016/0277-9536(92)90286-Y

24 Marteau TM, Bekker H. The development of a six-item short-form of the state scale of the Spielberger State-Trait Anxiety Inventory (STAI). *Br J Clin Psychol* 1992;**31 (Pt 3)**:301–6.

25 Herdman M, Gudex C, Lloyd A, *et al.* Development and preliminary testing of the new five-level version of EQ-5D (EQ-5D-5L). *Qual Life Res* 2011;**20**:1727–36. doi:10.1007/s11136-011-9903-x

26 Dugan E, Trachtenberg F, Hall MA. Development of abbreviated measures to assess patient trust in a physician, a health insurer, and the medical profession. *BMC Health Serv Res* 2005;**5**:1–7. doi:10.1186/1472-6963-5-64

27 Malterud K, Siersma VD, Guassora AD. Sample Size in Qualitative Interview Studies. *Qual Health Res* 2015;**26**:1753–60. doi:10.1177/1049732315617444

For peer review only

For peer review only - http://bmjopen.bmj.com/site/about/guidelines.xhtml

FIGURE LEGENDS

Figure 1. Study recruitment. YLST – Yorkshire Lung Screening Trial; YKST – Yorkshire Kidney Screening Trial; GP, general practice; LDCT, low- dose computed tomography; LLP, Liverpool Lung Project; PLCO, Prostate, Lung, Colorectal and Ovarian; USPSTF, US Preventive Services Task Force

Figure 2. Main study process map. YLST – Yorkshire Lung Screening Trial; T2 – second round of screening within YLST; LDCT – low dose CT; YKST – Yorkshire Kidney Screening Trial; CTA – clinical trials assistant; EOD – End of day report; LTHT – Leeds Teaching Hospitals Trust; PACS - Picture archiving and communication system; AAA – abdominal aortic aneurysm; CRIS – Clinical Record Interactive Search System

For peer review only

For peer review only - http://bmjopen.bmj.com/site/about/guidelines.xhtml

For peer review only

For peer review only - http://bmjopen.bmj.com/site/about/guidelines.xhtml

Figure 1. Study recruitment. YLST – Yorkshire Lung Screening Trial; YKST – Yorkshire Kidney Screening Trial; GP, general practice; LDCT, low- dose computed tomography; LLP, Liverpool Lung Project; PLCO, Prostate, Lung, Colorectal and Ovarian; USPSTF, US Preventive Services Task Force

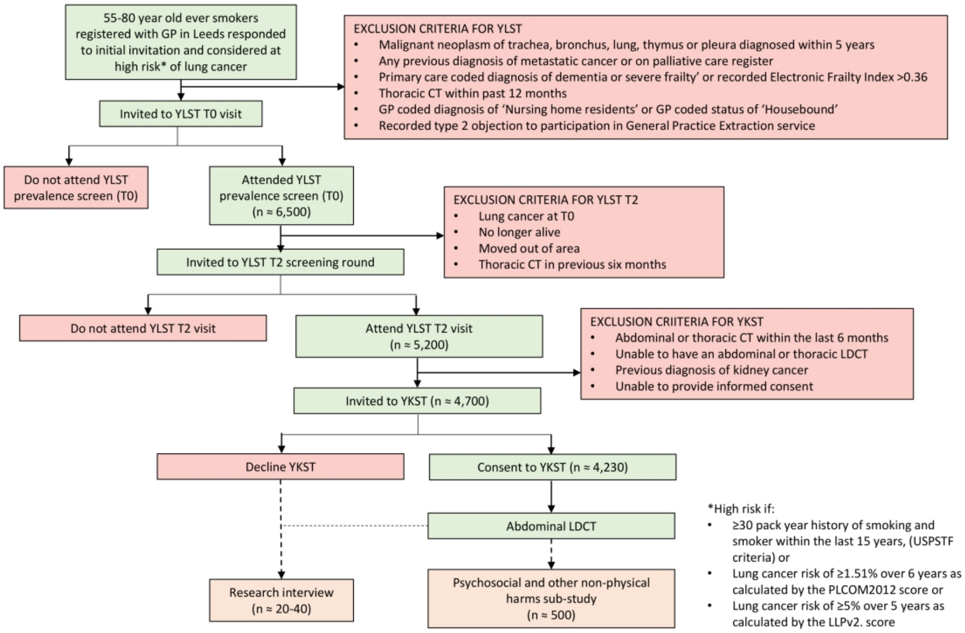

Figure 1. Study recruitment

275x190mm (300 x 300 DPI)

Figure 2. Main study process map. YLST – Yorkshire Lung Screening Trial; T2 – second round of screening within YLST; LDCT – low dose CT; YKST – Yorkshire Kidney Screening Trial; CTA – clinical trials assistant; EOD – End of day report; LTHT – Leeds Teaching Hospitals Trust; PACS - Picture archiving and communication system; AAA – abdominal aortic aneurysm; CRIS – Clinical Record Interactive Search System

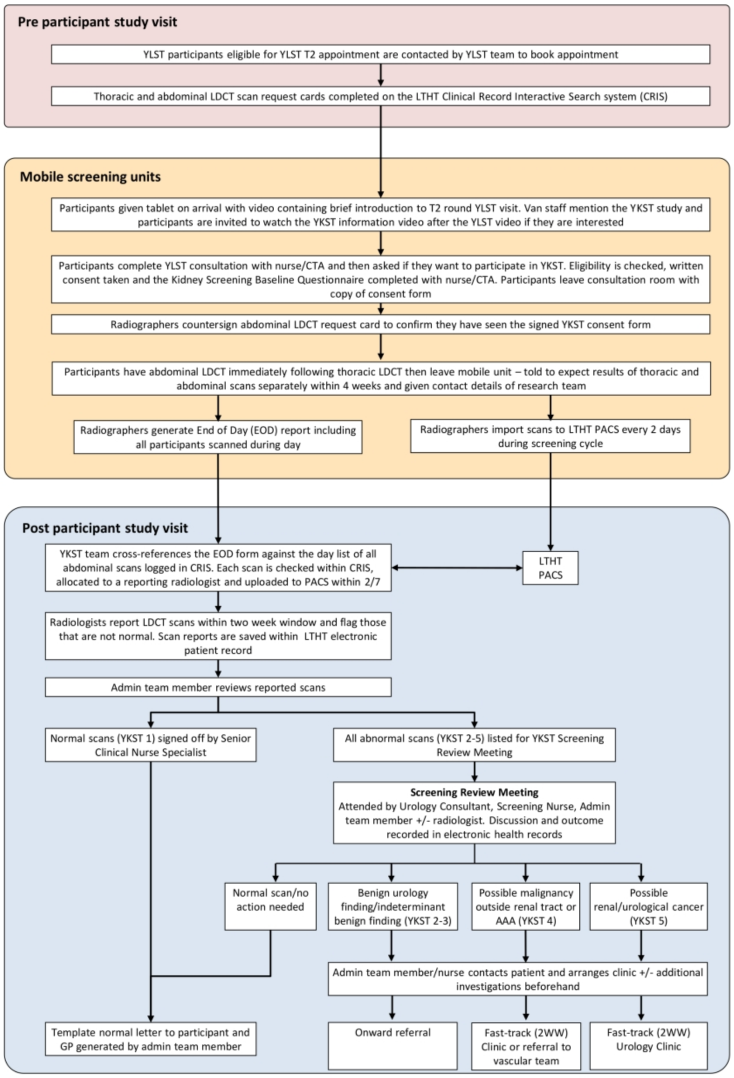

Figure 2. Main study process map

190x254mm (300 x 300 DPI)

The Yorkshire Kidney Screening Trial (YKST): protocol for a feasibility study of adding non-contrast abdominal CT scanning to screen for kidney cancer and other abdominal pathology within a trial of community-based CT screening for lung cancer

Journal:	*BMJ Open*
Manuscript ID	bmjopen-2022-063018.R1
Article Type:	Protocol
Date Submitted by the Author:	01-Aug-2022
Complete List of Authors:	Usher-Smith, Juliet; University of Cambridge, Department of Public Health and Primary Care<br>Pushpa-Rajah, Angela; University of Cambridge, Department of Surgery<br>Burge, Sarah; University of Cambridge, Cancer Research UK Urological Malignancies Programme, Department of Oncology<br>Burbidge, Simon; Leeds Teaching Hospitals NHS Trust<br>Cartledge, Jon; Saint James's University Hospital<br>Crosbie, Philip; The University of Manchester, Division of Infection, Immunity and Respiratory Medicine<br>Eckert, Claire; University of Leeds<br>Farquhar, Fiona; Leeds Teaching Hospitals NHS Trust<br>Hammond, David; Leeds Teaching Hospitals NHS Trust<br>Hancock, Neil; University of Leeds, School of Food Science and Nutrition<br>Iball, Gareth R; Leeds Teaching Hospitals NHS Trust<br>Kimuli, Michael; Saint James's University Hospital<br>Masson, Golnessa; University of Cambridge, Department of Public Health and Primary Care<br>Neal, Richard; University of Leeds<br>Rogerson, Suzanne; Leeds Teaching Hospitals NHS Trust<br>Rossi, Sabrina; University of Cambridge, Department of Surgery; Cambridge University Hospitals NHS Foundation Trust, Department of Urology<br>Sala, Evis ; University of Cambridge<br>Smith, Andrew; Leeds Teaching Hospitals NHS Trust<br>Sharp, Stephen; University of Cambridge, MRC Epidemiology Unit<br>Simmonds, Irene; University of Leeds<br>Wallace, Tom; Leeds Teaching Hospitals NHS Trust<br>Ward, Matthew; University of Leeds<br>CALLISTER, Matthew; Leeds Centre for Respiratory Medicine, St James's University Hospital<br>Stewart, Grant; University of Cambridge, ;
**Primary Subject Heading**:	Urology
Secondary Subject Heading:	Renal medicine, Oncology

Keywords:	Kidney tumours < ONCOLOGY, Urological tumours < ONCOLOGY, Computed tomography < RADIOLOGY & IMAGING, Urological tumours < UROLOGY

SCHOLARONE™
Manuscripts

The Yorkshire Kidney Screening Trial (YKST): protocol for a feasibility study of adding non-contrast abdominal CT scanning to screen for kidney cancer and other abdominal pathology within a trial of community-based CT screening for lung cancer

Juliet A Usher-Smith*[1], Angela Pushpa-Rajah*[2], Sarah W Burge[3], Simon Burbidge[4], Jon Cartledge[4], Philip AJ Crosbie[5], Claire Eckert[6], Fiona Farquhar[4], David Hammond[4], Neil Hancock[6], Gareth R Iball[4], Michael Kimuli[4], Golnessa Masson[1], Richard D Neal[7], Suzanne Rogerson[4], Sabrina H Rossi[2], Evis Sala[8], Andrew Smith[4], Stephen J Sharp[9], Irene Simmonds[6], Tom Wallace[4], Matthew Ward[6], Matthew E J Callister[4,6], Grant D Stewart[2]

[1]Department of Public Health and Primary Care, University of Cambridge

[2]Department of Surgery, University of Cambridge

[3]Department of Oncology, University of Cambridge

[4]Leeds Teaching Hospitals NHS Trust

[5]Division of Infection, Immunity and Respiratory Medicine, Faculty of Biology, Medicine and Health, University of Manchester, Manchester, UK

[6]Institute of Health Sciences, University of Leeds

[7]College of Medicine and Health, University of Exeter

[8]Department of Radiology, University of Cambridge

[9]MRC Epidemiology Unit, University of Cambridge

*Joint contributors

For peer review only - http://bmjopen.bmj.com/site/about/guidelines.xhtml

For peer review only

Corresponding author: Professor Grant D Stewart, Department of Surgery, University of Cambridge, Cambridge Biomedical Campus, CB2 0QQ, UK. Tel: 01223 769002. Email: gds35@cam.ac.uk

Word count: 3950

For peer review only

ABSTRACT

Introduction

Kidney cancer (renal cell cancer [RCC]) is the 7th commonest cancer in the UK. As RCC is largely curable if detected at an early stage and most patients have no symptoms, there is international interest in evaluating a screening programme for RCC. The Yorkshire Kidney Screening Trial (YKST) will assess the feasibility of adding non-contrast abdominal CT scanning to screen for RCC and other abdominal pathology within the Yorkshire Lung Screening Trial (YLST), a randomised trial of community-based CT screening for lung cancer.

Methods and analysis

In YLST, ever-smokers aged 55–80 years registered with a general practice in Leeds have been randomised to a Lung Health Check assessment, including a thoracic low-dose CT (LDCT) for those at high risk of lung cancer, or routine care. YLST participants randomised to the Lung Health Check arm who attend for the second round of screening at two years without a history of RCC or abdominal CT scan within the previous six months will be invited to take part in YKST. We anticipate inviting 4,700 participants. Those who consent will have an abdominal CT immediately following their YLST thoracic LDCT. A sub-set of participants and the health care workers involved will be invited to take part in a qualitative interview. Primary objectives are to: quantify the uptake of the abdominal CT, assess the acceptability of the combined screening approach and pilot the majority of procedures for a subsequent randomised controlled trial of RCC screening within lung cancer screening.

Ethics and Dissemination

For peer review only - http://bmjopen.bmj.com/site/about/guidelines.xhtml

YKST was approved by the North West-Preston Research Ethics Committee (21/NW/0021), and the Health Research Authority on 3/2/2021. Trial results will be disseminated at clinical meetings, in peer-reviewed journals and to policy makers. Findings will be made available to participants via the study website (www.YKST.org).

Trial registration numbers

NCT05005195 and ISRCTN18055040

For peer review only

For peer review only - http://bmjopen.bmj.com/site/about/guidelines.xhtml

Strengths and limitations

- YKST is the first study to investigate whether it is acceptable and feasible to combine lung and kidney cancer screening using non-contrast low-dose CT scanning.
- By nesting YKST within an on-going trial of lung cancer screening and performing the abdominal scan immediately after the lung scan, the additional abdominal screening can be conducted with very little additional cost or inconvenience to participants.
- The participants invited to take part in YKST are those who have already consented to take part in a lung screening trial and are considered at high risk of lung cancer so may not be representative of those invited to future cancer screening.
- A nested sub-study will enable assessment of psychological, social and financial harms, and dissatisfaction with health care.

For peer review only

For peer review only - http://bmjopen.bmj.com/site/about/guidelines.xhtml

INTRODUCTION

Kidney cancer (or renal cell cancer [RCC]) is the 7th most common cancer in the UK, and incidence is increasing[1]. As with other cancers, survival is strongly dependent on stage at diagnosis: five-year survival is 87% in stage I compared with 12% in stage IV[1]. Diagnosing RCC at an early stage is therefore central to improving survival[2]. A particular challenge for the diagnosis of RCC is that 60% of patients are asymptomatic, rising to 87% when considering only stage 1 cancers[3]. As a result, up to a third of patients present with incurable stage IV disease [2] and half of all patients developing the disease die from it[1].

The fact that RCC incidence is increasing, is largely curable if detected early, and most patients are asymptomatic at the time of diagnosis, has resulted in interest from both the scientific community and patient representatives for the development of an RCC screening programme. In particular, screening and early detection of RCC has been identified as a key research priority in three independent priority setting initiatives over the last five years[4–7]. However, despite three decades of interest in the topic, no definitive studies have been conducted and there remain a number of key uncertainties[8]. These include whether detecting RCC earlier would translate into reductions in mortality or lead to overdiagnosis and overtreatment and whether the benefits at population level would outweigh the potential physical, psychosocial and financial harms. Randomised controlled trials are, therefore, needed. Additionally, despite the increasing incidence, extrapolating from studies in the USA or Japan, the prevalence of RCC among middle-aged adults within the general population in the UK is estimated to be 0.21% (95% CI, 0.14–0.28%)[9]. This means that approximately 500 individuals would need to be screened to identify one person with a RCC unless screening was targeted towards higher-risk individuals[8].

For peer review only - http://bmjopen.bmj.com/site/about/guidelines.xhtml

The gold standard test for detecting and investigating renal masses is a contrast-enhanced abdominal computed tomography (CT) scan. It is not feasible to use a contrast-enhanced CT as a stand-alone screening test for RCC due to the relatively high radiation dose and cost, particularly given the low prevalence of RCC. However, using anon-contrast CT scan and combining that with the thoracic low-dose CT (LDCT) scans recommended in the USA in adults aged 50-80 years who have a 20 pack-year smoking history or have quit smoking within the past 15 years [10] and currently being reviewed by the UK National Screening Committee has been proposed[8]. Over 95% of deaths from RCC in the UK occur in those aged over 50 and the relative risk for RCC compared with never smokers is 1.35 for current smokers and 1.22 for ex-smokers. This combined approach would therefore reduce both the costs and radiation, while also targeting those at higher risk due to their age and smoking status.

The potential benefits of using CT to detect RCC have been seen in one of the randomised control trials of lung cancer screening in the USA[11] in which participants diagnosed with RCC within 12 months of the thoracic scan who had a reported abnormality in the upper abdomen had a significantly shorter median time to diagnosis that those without an abnormality in the upper abdomen. However, the thoracic LDCT used within lung cancer screening only includes the upper pole of the kidneys. Additionally, for any screening programme to be successful, eligible individuals need to take up the offer of screening. Previous research has shown that providing combined ‘one stop’ cancer screening

For peer review only - http://bmjopen.bmj.com/site/about/guidelines.xhtml

programmes is viewed positively by members of the public[12] and a survey of over 1000 individuals found that 95% would be "likely" or "very likely" to take up an abdominal CT for RCC screening if it was offered in addition to lung cancer screening[13]. These studies, however, report only intention, and not actual attendance, as no such screening programme currently exists. There are also no studies piloting the additional logistics required for such a combined screening programme.

The Yorkshire Lung Screening Trial (YLST) is a community based, lung screening programme that has recruited individuals who are current or ex-smokers, 55-80 years of age and at high risk of developing lung cancer as defined by the LLP_{v2} score[14], $PLCO_{M2012}$ [15] score or using the 2014 USPSTF criteria[16]. Participants are being invited back for a second thoracic LDCT after two years. Nested within YLST, the Yorkshire Kidney Screening Trial (YKST) will take advantage of this unique opportunity to assess the feasibility and acceptability of offering an additional non-contrast abdominal CT at the same time as the thoracic LDCT as a combined abdominal and lung cancer screening approach and to estimate other key uncertainties needed to inform a health economic analysis and subsequent randomised controlled trials within future screening programmes.

OBJECTIVES

The primary study objectives are:

1. To quantify the uptake of non-contrast abdominal CT to screen for RCC and other abdominal pathology as part of a combined screening modality with thoracic LDCT within a lung health check;

For peer review only - http://bmjopen.bmj.com/site/about/guidelines.xhtml

2. To assess the acceptability to patients of combined lung and RCC screening by non-contrast CT scanning;
3. To evaluate the logistics and acceptability to healthcare professionals involved in the combined lung and RCC screening pathway; and
4. To pilot the majority of procedures for a subsequent full-scale randomised controlled trial of RCC screening by non-contrast CT scanning within lung cancer screening.

The secondary study objectives are to estimate:

1. The prevalence of renal masses and RCC found on non-contrast CT screening in an appropriate age (55-80y) and risk group (smokers and ex-smokers);
2. The stage distribution of RCC identified through non-contrast CT screening;
3. The prevalence of incidental renal findings on non-contrast CT scanning;
4. The prevalence of non-renal findings on non-contrast CT scanning; and
5. The incidence of RCC in the upper pole of the kidney over sequential non-contrast CT scans.

OUTCOME MEASURES

The primary outcome measures are:

1. The proportion of individuals invited to have an additional abdominal CT while attending a second round of lung cancer screening who take up the offer of the abdominal CT;
2. The acceptability to participants of combined lung and RCC screening by non-contrast CT scanning;

For peer review only

3. The acceptability to healthcare professionals involved in the combined screening approach; and
4. The additional time required for the combined screening approach.

The secondary outcome measures are:

1. The proportion of participants found to have a renal mass or RCC to provide an estimate of the prevalence of RCC found on non-contrast CT screening in 55-80y smokers and ex-smokers;
2. The stage distribution of RCC identified through non-contrast CT screening;
3. The proportion of participants found to have incidental renal findings on non-contrast CT scanning;
4. The proportion of participants with non-renal findings on non-contrast CT scanning; and
5. The proportion of RCCs found on the upper pole of participants at the second thoracic screening round who did not have them in the baseline round, to estimate the incidence of RCC over sequential non-contrast CT scans.

Data will also be collected on further investigations, procedures and management of findings identified on abdominal CT to estimate the individual and health system burden of incidental findings and on the agreement of radiologists reporting the scans and the abdominal CT scan dose and quality to assess the safety. We will also collect long term (10 year) follow-up data on RCC and other abdominal pathology and apply for CAG approval to obtain data on RCC amongst participants within YLST who were not invited to take part in YKST.

For peer review only - http://bmjopen.bmj.com/site/about/guidelines.xhtml

METHODS AND ANALYSIS

Study design

YKST is a non-randomised feasibility study of adding an abdominal CT scan to the thoracic LDCT offered to participants two years after recruitment into the Yorkshire Lung Screening Trial (YLST)[17].

Participants and Recruitment

Participant recruitment is detailed in Figure 1. Participants will be recruited from those attending the second (T2) round of screening within YLST from May 2021 to October 2022. Full details of YLST are published elsewhere[17]. In brief, YLST is a two-arm (1:1) implementation study using a single-consent Zelen's randomised controlled design with participants randomised to a Lung Health Check or usual care. Participants randomised to the intervention arm are invited to contact a telephone line for a lung cancer risk assessment. Those at high risk of lung cancer are offered a Lung Health Check appointment at a mobile unit sited in convenient community locations, including LDCT screening for lung cancer. The YLST screening programme includes a baseline visit (T0), where participants undergo baseline measurements of height and weight, spirometry (pre-SARS-CoV-2 pandemic), oxygen saturation and exhaled carbon monoxide alongside a smoking cessation intervention and a thoracic LDCT, and a second visit two years later (T2), where participants are offered a further thoracic LDCT.

For peer review only - http://bmjopen.bmj.com/site/about/guidelines.xhtml

Eligibility for YLST is detailed in Figure 1. All those not diagnosed with lung cancer or any other metastatic cancer following the YLST baseline visit (T0) are invited back for T2 between May2021 and October 2022. The exclusion criteria for YKST at that point are:

- Abdominal or thoracic CT within the last 6 months
- Unable to have an abdominal or thoracic LDCT
- Previous diagnosis of RCC
- Unable to provide informed consent

Invitation process, consent and baseline data collection

The study processes are shown in Figure 2. Participants who attend the mobile van for their YLST T2 visit will be informed of YKST on the van. They will be invited to view the YLST T2 Patient Information video, followed directly by the YKST information video. The YKST video explains the context of the study and the benefits and harms of the additional abdominal CT, including an estimate that in about 5 out of 1000 eligible people the scan may show evidence of RCC, the uncertainty over whether detecting cancers in this way reduces deaths from RCC, the risks associated with the radiation dose and overdiagnosis and the potential to cause anxiety and worry. Participants are also provided with a written participant information sheet covering the same information (Supplementary File 1). A YLST consultation will follow, at the end of which participants will be asked if they would like to take part in YKST. Translation services are offered to patients where required. Eligibility will be checked and fully informed written consent obtained. As part of this consent, participants will consent to allowing the YKST research team access to their medical records.

For peer review only

Participants who consent, as well as those who decline the additional scan, will be invited to take part in a qualitative interview. Participants who consent to being contacted about an interview, will be asked to provide their contact details. A separate participant information sheet and consent form will be sent to them and they will be asked to contact a qualitative researcher to arrange an interview.

After providing informed consent, participants will complete a short YKST baseline questionnaire asking whether they have a diagnosis of diabetes or hypertension, whether they take antihypertensive medication, if they have a family history of kidney or pancreatic cancer and their average weekly alcohol consumption. Sociodemographic data (age, sex, socioeconomic status, educational level) and height, weight and smoking status will be obtained from data collected in YLST.

Participants will then be shown to a separate room on the van to have the YLST thoracic LDCT, followed immediately by the YKST abdominal CT. To ensure that only those participants who have consented to YKST receive the additional abdominal CT, radiographers will only perform the abdomen scan for those participants who have i) signed the YKST consent form, and ii) from whom they have received a YKST LDCT request card.

CT scanning protocol

The scanning protocol for the non-contrast abdominal CT will be based on the protocol used for kidney ureter and bladder (KUB) scans within Leeds Teaching Hospital Trust (LTHT) and will be reviewed and monitored by the LTHT medical physics team to ensure that the lowest possible dose allowing interpretable images is used for the YKST abdominal images. A 64-

For peer review only - http://bmjopen.bmj.com/site/about/guidelines.xhtml

channel (or higher) mobile multidetector CT will be used throughout the study. Participants will lie supine on the CT table with arms above their head and thorax and abdomen in the midline of the scanner. Subject comfort will be optimised and maximal inspiration rehearsed prior to the scan to minimise motion during the CT. Imaging will then be performed during suspended maximal inspiration with the standard scanogram used to localise the start and end positions of the scan. No intravenous contrast material will be administered. The kidneys will be scanned in their entirety in a single craniocaudal acquisition and transaxial images of 1mm thickness will be generated, with further reconstructions as necessary. Radiation exposures will be kept as low as possible whilst maintaining good image quality. The average CT dose index (CTDIvol) and Dose Length Product (DLP) for 70-80kg patients will be monitored to ensure they closely match the current typical values of 5mGy and 110mGycm respectively from LTHT scanners. The x-ray tube current (mAs) settings will be automatically varied by the scanner according to participant body habitus. The CT images will then be transferred from the mobile unit to LTH PACS system within 2 days.

CT scan reporting

A team of LTHT uro-radiology consultants will report the abdominal CT scans. They will receive them through the LTHT (PACS) systems and will report them within two weeks of the date of the scan. Scans and reports will be stored on the LTHT electronic patient record (PPM+). The time taken to access and generate the report for these scans will be collected as part of a process evaluation.

The abdominal CT scans will be classified according to one of the five categories below:

For peer review only - http://bmjopen.bmj.com/site/about/guidelines.xhtml

- YKST1 - Normal
- YKST2 - Benign urological finding
- YKST3 - Indeterminate benign finding (i.e. cholecystitis/pancreatitis)
- YKST4 - Possible malignancy outside renal tract or abdominal aortic aneurysm (AAA)
- YKST5 - Possible renal/urological cancer

Normal scan reports (YKST1) will be reviewed and signed off by a senior clinical nurse specialist. A letter will be sent to the patient and their GP, explaining that their scan was normal and that there are no further actions required.

All scans not reported as normal, as well as any normal LDCT scans with discordant reports after second reads (see quality assurance details below), will be reviewed in a screening review meeting (SRM). SRMs will take place twice weekly and will be attended by a consultant urologist with an interest in renal cancer, a senior clinical nurse specialist and a clinical trials administrator. The administrative team will record the agreed outcome and communicate the results to participants and their GPs according to Table 1. Participants will be able to contact the YKST team via the YKST website (www.ykst.org) and the YKST phone number.

For peer review only - http://bmjopen.bmj.com/site/about/guidelines.xhtml

Table 1: Outcomes from Screening Review Meeting

Outcome	Reason	Action	Communication
Normal	No abnormal findings – No Action required	Discharge	Patients and GPs sent letter communicating result
Benign urological and non-urological findings	Benign findings - No Action required	Discharge	Patients and GPs sent letter communicating benign findings and that no further action is required
Indeterminate benign finding	Indeterminate finding requiring further elective investigations	Referral to appropriate speciality coordinated by Consultant Urologist and their delegates. Further tests requested as appropriate following recommendations by radiologist.	YKST lead nurse telephones patients (except for adrenal referrals where referral team contacts patients immediately after the referral is made).<br><br>Patients then contacted by relevant speciality administrative team scheduling appointment and copy sent to GP.
Possible malignancy outside the renal tract or AAA	Abnormality requiring immediate further investigation for possible abdominal cancer or AAA	Fast Track 2 week wait appropriate speciality coordinated by Consultant Urologist and their delegates.	YKST lead nurse contacts patients explaining findings, need for further investigations or onward referral.<br><br>Patients then contacted by relevant speciality administrative team scheduling appointment and copy sent to GP.
Possible renal/urological cancer	Abnormality requiring immediate further investigation for possible renal or urological cancer	During YKST scan review meeting: Consultant Urologist or delegates request contrast scan, refer patient to urology MDT, and assign them to fast track 2 week wait pathway.	YKST lead nurse telephones patients explaining findings and need for further investigations.<br><br>Patients then contacted by relevant speciality administrative team scheduling appointment and copy sent to GP.

AAA – abdominal aortic aneurysm

For peer review only - http://bmjopen.bmj.com/site/about/guidelines.xhtml

Quality Assurance

Ten percent of all normal scans (YKST 1) will be selected at random, re-reported by a different radiologist, and categorised as YKST 1–5 in a second report. The quality of the scans will be assessed both qualitatively using a Likert score from 1 (poor) to 5 (excellent) and quantitatively by selecting a region of interest (ROI). The Likert scale will be recorded by the radiologists for all scans. The ROI assessment will only be performed on the 10% of scans that are second read and will be reported in an addendum to the scan report.

Qualitative interviews

The interviews will take place over the telephone or video call. The interview schedule will be informed by the Theoretical Framework of Acceptability[18] and explore participants' views on the acceptability of the information provided, the consent process, their thoughts on the combined screening approach, and their reasons for accepting or declining the abdominal scan. The interviews will also explore any psychological harm or anxiety that may be experienced by taking part in this combined screening approach. Health care professionals who are involved in the study will also be invited to take part in an interview to assess the acceptability of the combined screening approach to staff members. All interviews will be recorded and transcribed.

Follow-Up

To capture the potential downstream harms of the abdominal CT scan, the medical notes of all participants who had an abnormal finding on the abdominal CT will be reviewed six months after the scan by the study team; to identify all investigations, procedures, complications, diagnoses and management arising from findings on the abdominal CT. For

For peer review only - http://bmjopen.bmj.com/site/about/guidelines.xhtml

participants who move out of the study area, all reasonable efforts will be made to determine what their outcome was. Incidental Findings will be divided into serious and non-serious based on whether or not they represent a condition which carries a real prospect of seriously threatening life span, or of having a substantial impact on major body functions or quality of life[19]. The classification of findings will be performed by two clinicians independently based on the clinical information within the patient electronic health records and the list of potentially serious / non-serious incidental findings developed in a previous study for abdominal MRI scans based on consultations with radiologists, review of the literature and the German National Cohort's list of imaging incidental findings[19] and consultation with the clinicians within the research team. Agreement between the two clinicians will be reported by calculating the percentage of findings for which both clinicians agreed on the initial classification. Any discrepancies will be reviewed and discussed at a consensus meeting.

Long term follow-up will take place between months 20 and 120, and will include: number of kidney and other upper abdominal cancers detected and histological subtype, pathological tumour stage and grade; number and details of non-cancer findings; cancer stage at diagnosis; treatments received; date and cause of death.

Psychosocial and other non-physical harms sub-study

A sub-set of approximately 500 participants consisting of all those who have an abnormal CT scan report (YKST 2-5) between March 2022 and October 2022 and a random sample of one third of those with normal scans (YKST 1) recruited within the same time period will be sent a short questionnaire three months and six months after the scan to evaluate outcomes in

For peer review only - http://bmjopen.bmj.com/site/about/guidelines.xhtml

relation to psychological, social and financial harms, and dissatisfaction with health care. Questionnaires will be sent by post, with participants having the option to complete the questionnaire online and one reminder will be sent two weeks after each questionnaire to reduce non-response bias. The questionnaire will include validated measures where possible, including the Psychological Consequences Questionnaire (PCQ)[20], the Short form of the Spielberger State Trait Anxiety Inventory (STAI)[21], the EQ-5D-5L[22], and a single question asking how participants would rate their general health now compared to before they were invited to take part in YKST. The financial consequences of having the scan will be measured using five questions from a previous study[19] and satisfaction with healthcare using the abbreviated measure to assess trust in the medical profession[23]. The questionnaire will also assess participant satisfaction with the information they received and whether they felt they had had sufficient time and information to make the decision whether or not to accept the scan.

Withdrawal of consent

If participants wish to withdraw from the study no further data will be collected on them, though we will keep all data collected to that point. All patient withdrawals will be recorded.

Safety

Adverse events occurring between the time the participants enter the mobile van for their T2 visit and the time that their final result letter is written to them and they are discharged from the study will be recorded and reported in line with Good Clinical Practice.

Sample size

For peer review only - http://bmjopen.bmj.com/site/about/guidelines.xhtml

The maximum sample size is limited to those participants who attend for their YLST T2 visit. Approximately 6500 participants were recruited into YLST and it is estimated that 80% of those will attend for the T2 visit. Recruitment began two months into T2 on 10 May 2021 and will run until 31 October 2022. Approximately 4,700 individuals will therefore be eligible for inclusion into YKST. If 80-90% of those take up the additional screening, it will be possible to measure the proportion taking up the additional scan, the primary quantitative outcome of this study, to within 1%. For the qualitative sub-study, the principles of information power[24] will be used to decide when to cease data collection but we anticipate interviewing up to 40 participants. We will purposefully sample participants with the aim to include approximately 20 who accept the additional scan and 20 who do not, with a range of ages, sex, ethnicity and socioeconomic status. For the qualitative interviews with healthcare professionals, there are approximately 10 closely involved in the screening process and all will be approached.

Data analysis

Primary outcomes

We will report the proportion of the population attending the T2 screening round who i) are eligible to take part in YKST; ii) are invited to take part in YKST; iii) consent to the additional scan within YKST; iv) decline taking part in YKST. We will also report these proportions by age, sex, smoking status, ethnicity and socio-economic status, and compare those invited who accept and undergo the abdominal CT scan between demographic subgroups. The additional time required at each stage (obtaining consent, performing, reporting and reviewing the scans, and feeding back the results to participants) of the combined screening approach will be reported. Qualitative data evaluating the acceptability of the combined

For peer review only - http://bmjopen.bmj.com/site/about/guidelines.xhtml

screening approach will be analysed using Framework analysis, guided by the Theoretical Framework of Acceptability[18]. Each transcript will be read by at least two members of the study team with other members of the study team reading some of the transcripts and contributing to discussions about the overall findings.

Secondary outcomes

Descriptive summaries of secondary outcomes will be reported. All clinical outcomes will be based on the final diagnosis obtained from the six month follow-up data. When reporting the prevalence and stage distribution of RCC, we will present data among the participants who had the abdominal CT as well as among those from the baseline round of scanning in YLST who either had a renal mass identified in that baseline (T0) lung LDCT or staging investigations for any lung lesions identified and so would have had their full kidneys imaged.

Patient and Public involvement

Two members of the public were involved in the design of this study and contributed to the research proposal prior to submission for funding. They have also commented on all participant facing documentation and continue to contribute to the study as members of the Independent Trial Steering Committee.

ETHICS AND DISSEMINATION

This study was granted approval by the North West - Preston Research Ethics Committee (reference 21/NW/0021), and the Health Research Authority on 3rd February 2021. It has been adopted onto the National Institute for Health Research trial portfolio (reference

For peer review only - http://bmjopen.bmj.com/site/about/guidelines.xhtml

290336). The University of Leeds is the sponsor and together with LTHT acts as joint data controller. The study has been registered on the International Standard Randomised Controlled Trial Number (ISRCTN) (reference ISRCTN18055040) and the National Institutes of Health ClinicalTrials.gov database (reference NCT05005195). The trial will have three committees providing oversight: the Trial Management Group (TMG), the Independent Data Monitoring Committee (IDMC) and an Independent Trials Steering Committee (TSC). The TMG will meet on a monthly basis, and will consist of the co-ordinating team based in Cambridge, members of the YKST team based in Leeds as well as the YLST principal investigator, data manager, project manager and lead nurse. The TMG will provide regular monitoring of the trial and provide clinical, scientific and practical advice. The IDMC will meet once or twice a year and will monitor patient safety as well as interim data. The TSC will meet once or twice a year and will provide overall oversight for the trial. The independent members of the IDMC and TSC will include experts in the field of cancer screening, radiology, renal cancer and statistics. The TSC will also include at least one a patient/public representative.

Findings from the study will be reported in open-access papers in peer-reviewed journals and presented at national and international conferences. We will also provide a lay summary of the findings on the study website (www.YKST.org).

DISCUSSION

As the first study of its kind, YKST will assess the feasibility and acceptability of a combined abdominal and lung cancer screening approach and estimate other key uncertainties needed to inform a health economic analysis and future randomised controlled trials.

For peer review only - http://bmjopen.bmj.com/site/about/guidelines.xhtml

Nesting YKST within an on-going randomised lung cancer screening trial also provides a unique opportunity to generate the first cohort of participants invited to undergo screening for RCC. Although limited to assessing uptake and acceptability among participants who have already accepted screening for lung cancer and not large enough on its own to enable precise estimates of prevalence of RCC or an assessment of whether screening for RCC reduces RCC mortality, this cohort will be a valuable foundation for future research.

Acknowledgements

The authors thank the patient and public representations who have contributed to this study, Phil Alsop and Philip Dondi. They also thank the YLST study team and members of the YKST IDMC (Paul Nathan (Chair), Vicky Goh, Damian Hanbury and Akhtar Nasim) and TSC (Peter Sasieni (Chair), Jonathan Mant, David Nicol, Robert Rintoul, Katie Robb and Jo Waller).

Authors' contributions

Conceptualisation: GDS, JAU. Design: GDS, MEJC, JAU, SWB, RDN, SR, SHR, ES, TW, AS, GRI, SB, JC. Draft: AP, GDS, JAU. Revision: SWB, SB, JC, PAJC, CE, FF, DH, NH, GRI, MK, GM, RDJ, SR, SHR, ES, AS, SJS, IS, TW, MW, MEJC, GDS, JAU, AP.

Funding

This study is funded by Yorkshire Cancer Research grant number L403C. The qualitative sub-study is funded by a grant from Kidney Cancer UK. GDS is supported by The Mark Foundation for Cancer Research, the Cancer Research UK Cambridge Centre [C9685/A25177] and NIHR Cambridge Biomedical Research Centre (BRC-1215-20014). PAJC

For peer review only - http://bmjopen.bmj.com/site/about/guidelines.xhtml

is supported by the Manchester National Institute for Health Research Manchester Biomedical Research Centre (IS-BRC-1215-20007). The views expressed are those of the author(s) and not necessarily those of the NIHR or the Department of Health and Social Care.

Patient consent for publication

Not required

Data sharing

In order to meet our ethical obligation to responsibly share data generated by clinical trials, YKST operates a transparent data sharing request process. Anonymous data will be available for request once the study has published the final proposed analyses. Researchers wishing to use the data will need to complete a Request for Data Sharing form describing a methodologically sound proposal. The form will need to include the objectives, what data are requested, timelines for use, intellectual property and publication rights, data release definition in the contract and participant informed consent etc.. A Data Sharing Agreement from the Sponsor may also be required.

Competing interests

All authors have completed the Unified Competing Interest form at www.icmje.org/coi_disclosure.pdf (available on request from the corresponding author). GDS has received educational grants from Pfizer, AstraZeneca, and Intuitive Surgical; consultancy fees from Pfizer, Merck, EUSA Pharma, and CMR Surgical; travel expenses from Pfizer; and speaker fees from Pfizer.

For peer review only - http://bmjopen.bmj.com/site/about/guidelines.xhtml

All other authors declare that (1) they have no support from or relationships with companies that might have an interest in the submitted work in the previous 3 years; (2) their spouses, partners, or children have no financial relationships that may be relevant to the submitted work; and (3) they have no non-financial interests that may be relevant to the submitted work.

The corresponding author has the right to grant on behalf of all authors and does grant on behalf of all authors, an exclusive licence on a worldwide basis to the BMJ Publishing Group Ltd and its Licensees to permit this article (if accepted) to be published in BMJ editions and any other BMJPGL products and sub-licences to exploit all subsidiary rights, as set out in our licence (http://resources.bmj.com/bmj/authors/checklists-forms/licence-for-publication).

The corresponding author affirms that the manuscript is an honest, accurate, and transparent account of the study being reported; that no important aspects of the study have been omitted; and that any discrepancies from the study as planned (and, if relevant, registered) have been explained.

For peer review only

REFERENCES

1 Kidney cancer statistics | Cancer Research UK. https://www.cancerresearchuk.org/health-professional/cancer-statistics/statistics-by-cancer-type/kidney-cancer (accessed 30 May 2019).

2 Bhindi B, Lohse CM, Mason RJ, *et al.* Are We Using the Best Tumor Size Cut-points for Renal Cell Carcinoma Staging? *Urology* 2017;**109**:121–6.

doi:10.1016/j.urology.2017.04.010

3 Vasudev NS, Wilson M, Stewart GD, *et al.* Challenges of early renal cancer detection: Symptom patterns and incidental diagnosis rate in a multicentre prospective UK cohort of patients presenting with suspected renal cancer. *BMJ Open* 2020;**10**:1–9. doi:10.1136/bmjopen-2019-035938

4 Rossi SH, Blick C, Handforth C, *et al.* Essential Research Priorities in Renal Cancer: A Modified Delphi Consensus Statement. *Eur Urol Focus* Published Online First: February 2019. doi:10.1016/j.euf.2019.01.014

5 Rossi SH, Fielding A, Blick C, *et al.* Setting Research Priorities in Partnership with Patients to Provide Patient-centred Urological Cancer Care. *Eur Urol* 2019;**75**:891–3. doi:10.1016/j.eururo.2019.03.008

6 Rini B, Abel EJ, Albiges L, *et al.* Summary from the Kidney Cancer Association's Inaugural Think Tank: Coalition for a Cure. *Clin Genitourin Cancer* 2021;**19**:167–75. doi:10.1016/J.CLGC.2020.10.005

7 Jones J, Bhatt J, Avery J, *et al.* The kidney cancer research priority-setting partnership: Identifying the top 10 research priorities as defined by patients, caregivers, and expert clinicians. *Can Urol Assoc J* 2017;**11**:379–87. doi:10.5489/cuaj.4590

8 Usher-Smith J, Simmons R, Rossi S, *et al.* Current evidence on screening for renal cancer. *Nat Rev Urol* Published Online First: 2020. doi:10.1016/B978-0-323-47873-1.00100-5

9 Rossi SH, Hsu R, Blick C, *et al.* Meta-analysis of the prevalence of renal cancer detected by abdominal ultrasonography. *Br J Surg* 2017;**104**:648–59. doi:10.1002/bjs.10523

10 Krist AH, Davidson KW, Mangione CM, *et al.* Screening for Lung Cancer: US Preventive

For peer review only

For peer review only - http://bmjopen.bmj.com/site/about/guidelines.xhtml

Services Task Force Recommendation Statement. *JAMA - J Am Med Assoc* 2021;**325**:962–70. doi:10.1001/jama.2021.1117

11 Pinsky PF, Dunn B, Gierada D, *et al.* Incidental renal tumours on low-dose CT lung cancer screening exams. *J Med Screen* 2017;**24**:104–9. doi:10.1177/0969141316657115

12 Bobridge A, Price K, Gill TK, *et al.* Influencing cancer screening participation rates-providing a combined cancer screening program (a 'One Stop' shop) could be a potential answer. *Front Oncol* 2017;**7**:1–7. doi:10.3389/fonc.2017.00308

13 Harvey-Kelly LLW, Harrison H, Rossi SH, *et al.* Public attitudes towards screening for kidney cancer: an online survey. *BMC Urol* 2020;:1–10. doi:10.1186/s12894-020-00724-0

14 Cassidy A, Myles JP, Van Tongeren M, *et al.* The LLP risk model: an individual risk prediction model for lung cancer. *Br J Cancer* 2008;**98**:270. doi:10.1038/SJ.BJC.6604158

15 Tammemägi MC, Katki HA, Hocking WG, *et al.* Selection Criteria for Lung-Cancer Screening. *N Engl J Med* 2013;**368**:728–36. doi:10.1056/nejmoa1211776

16 Moyer VA. Screening for lung cancer: U.S. preventive services task force recommendation statement. *Ann Intern Med* 2014;**160**:330–8. doi:10.7326/M13-2771

17 Crosbie PA, Gabe R, Simmonds I, *et al.* Yorkshire Lung Screening Trial (YLST): protocol for a randomised controlled trial to evaluate invitation to community-based low-dose CT screening for lung cancer versus usual care in a targeted population at risk. *BMJ Open* 2020;**10**:e037075. doi:10.1136/bmjopen-2020-037075

18 Sekhon M, Cartwright M, Francis JJ. Acceptability of health care interventions: A

For peer review only

For peer review only - http://bmjopen.bmj.com/site/about/guidelines.xhtml

theoretical framework and proposed research agenda. *Br J Health Psychol* 2018;:519–31. doi:10.1111/bjhp.12295

19 Gibson LM, Littlejohns TJ, Adamska L, *et al.* Impact of detecting potentially serious incidental findings during multi-modal imaging [version 3; referees: 2 approved, 1 approved with reservations]. *Wellcome Open Res* 2018;**2**. doi:10.12688/wellcomeopenres.13181.3

20 Cockburn J, De Luise T, Hurley S, *et al.* Development and validation of the PCQ: a questionnaire to measure the psychological consequences of screening mammography. *Soc Sci Med* 1992;**34**:1129–34. doi:10.1016/0277-9536(92)90286-Y

21 Marteau TM, Bekker H. The development of a six-item short-form of the state scale of the Spielberger State-Trait Anxiety Inventory (STAI). *Br J Clin Psychol* 1992;**31 (Pt 3)**:301–6.

22 Herdman M, Gudex C, Lloyd A, *et al.* Development and preliminary testing of the new five-level version of EQ-5D (EQ-5D-5L). *Qual Life Res* 2011;**20**:1727–36. doi:10.1007/s11136-011-9903-x

23 Dugan E, Trachtenberg F, Hall MA. Development of abbreviated measures to assess patient trust in a physician, a health insurer, and the medical profession. *BMC Health Serv Res* 2005;**5**:1–7. doi:10.1186/1472-6963-5-64

24 Malterud K, Siersma VD, Guassora AD. Sample Size in Qualitative Interview Studies. *Qual Health Res* 2015;**26**:1753–60. doi:10.1177/1049732315617444

For peer review only - http://bmjopen.bmj.com/site/about/guidelines.xhtml

FIGURE LEGENDS

Figure 1. Study recruitment. YLST – Yorkshire Lung Screening Trial; YKST – Yorkshire Kidney Screening Trial; GP, general practice; LDCT, low- dose computed tomography; LLP, Liverpool Lung Project; PLCO, Prostate, Lung, Colorectal and Ovarian; USPSTF, US Preventive Services Task Force

Figure 2. Main study process map. YLST – Yorkshire Lung Screening Trial; T2 – second round of screening within YLST; LDCT – low dose CT; YKST – Yorkshire Kidney Screening Trial; CTA – clinical trials assistant; EOD – End of day report; LTHT – Leeds Teaching Hospitals Trust; PACS - Picture archiving and communication system; AAA – abdominal aortic aneurysm; CRIS – Clinical Record Interactive Search System

For peer review only

For peer review only - http://bmjopen.bmj.com/site/about/guidelines.xhtml

For peer review only

For peer review only - http://bmjopen.bmj.com/site/about/guidelines.xhtml

Figure 1. Study recruitment. YLST – Yorkshire Lung Screening Trial; YKST – Yorkshire Kidney Screening Trial; GP, general practice; LDCT, low- dose computed tomography; LLP, Liverpool Lung Project; PLCO, Prostate, Lung, Colorectal and Ovarian; USPSTF, US Preventive Services Task Force

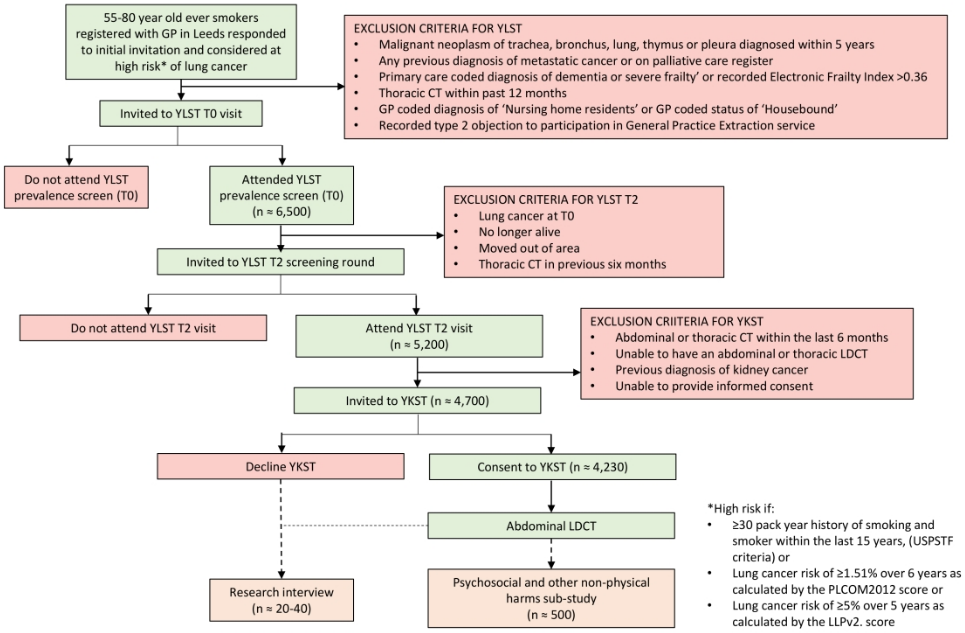

Figure 1. Study recruitment

275x190mm (300 x 300 DPI)

Figure 2. Main study process map. YLST – Yorkshire Lung Screening Trial; T2 – second round of screening within YLST; LDCT – low dose CT; YKST – Yorkshire Kidney Screening Trial; CTA – clinical trials assistant; EOD – End of day report; LTHT – Leeds Teaching Hospitals Trust; PACS - Picture archiving and communication system; AAA – abdominal aortic aneurysm; CRIS – Clinical Record Interactive Search System

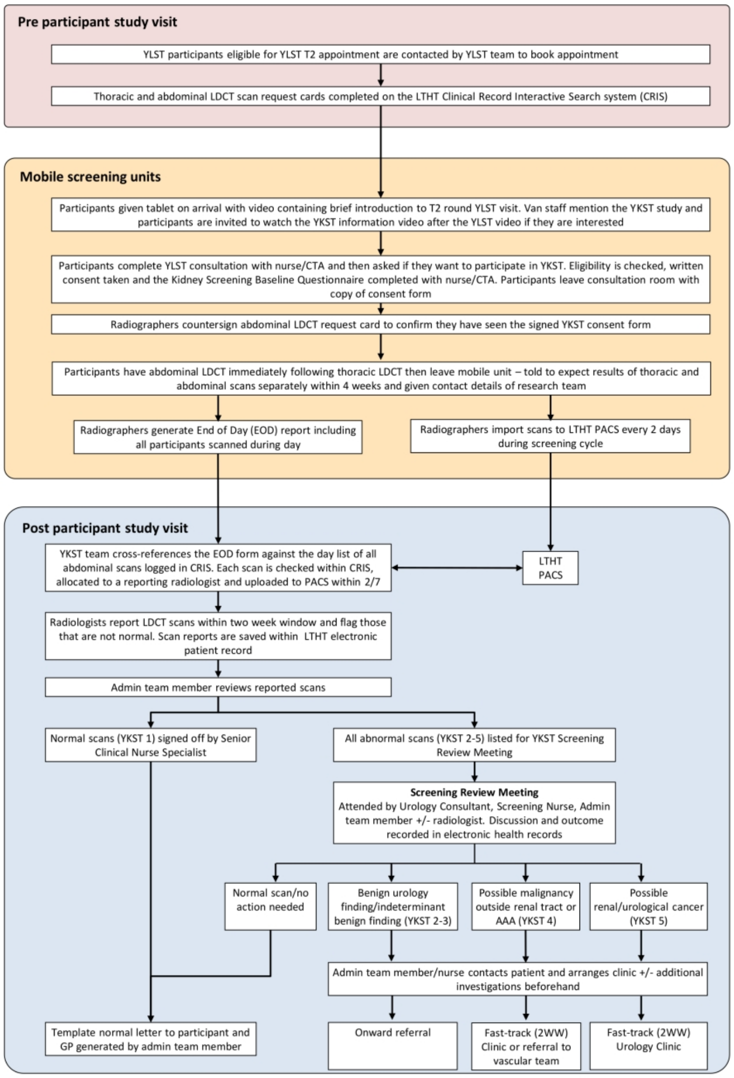

Figure 2. Main study process map

190x254mm (300 x 300 DPI)

Supplementary File 1

Yorkshire Kidney Screening Trial
Patient Information Sheet

For peer review only

Thank you for your interest in taking part in the Yorkshire Kidney Screening Trial today and having an additional CT scan of your kidneys at the same time as your lung scan. This leaflet gives you more information about the study. A video with the same information as this leaflet is available if you would prefer this.

What will happen if I decide to take part?
If you would like to, you can have an additional CT scan of your kidneys at the same time as your lung scan today. Whilst going through the lung health check questionnaire with you, a member of the research team will ask if you are interested in also being part of the kidney screening study. If you are, the researcher will go through the information in this leaflet and check you understand what will happen and are happy to take part. We will then ask you to sign a consent form and answer some additional questions about your kidney health. When you have your lung CT scan, you will also have the extra scan of your kidneys. This will happen immediately following the lung CT and take an extra 10-15 seconds. You will not need to change position. The scan is still pain-free and you will not need an injection. Trained staff will be present and they will talk you through what is happening. The whole lung and kidney health checks including the scans will still take less than one hour.

You will also be asked whether you would be happy to potentially be contacted by a member of the research team at a later date to take part in a phone or video interview. The purpose of the interview will be to talk about how you felt being offered an additional kidney CT scan and why you did or did not choose to have the extra scan. If enough people have already taken part in these interviews by the time you come for your lung health check, you may not be contacted by the research team even if you say you would be happy to take part in an interview.

You may also receive a short questionnaire 3 months and 6 months after your scan. Anyone who does not have a normal scan as well as a random sample of those with normal scans will be sent a questionnaire. This questionnaire will include questions about how you have felt since having the Kidney CT scan, to help us understand how having this scan may affects people's lives. The questionnaire will also ask you about your experience of taking part in the study to help design any future screening programmes. Each questionnaire should take no more than 10 minutes to complete and will be sent in the post with a pre-paid envelope so that you can easily return it to the study team. There will also be the option to complete the questionnaire online if you prefer that. You will be given a study ID if you decide to complete the

For peer review only - http://bmjopen.bmj.com/site/about/guidelines.xhtml

questionnaire online, so there will be no need for you to provide your name, email address or any identifiable information. We will not save any data on IP addresses. Details will be included on the paper version along with a telephone number to call if you have any questions about the questionnaire or need help completing it.

Why are you offering me a Kidney CT scan?

The kidney CT scan is looking for any changes in your kidneys that might need treatment. One of the particular things we are looking for are any early signs of kidney cancer. Checking for cancer in this way is called screening. You may have heard of screening for other types of cancer such as breast cancer or bowel cancer.

Kidney cancer is the 8th most common cancer in Yorkshire. It has a poor survival rate, with only 6 out of 10 patients diagnosed with kidney cancer still alive after 5 years. This is partly because many people with kidney cancer don't have any symptoms. Kidney cancer is often not diagnosed until the disease has passed the point at which we can easily cure it. Screening for kidney cancer has the potential to pick up these cancers earlier and increase the number of people who can be cured.

There are a variety of tests which could be used to screen for kidney cancer. These include a CT scan of the kidneys. It would not be appropriate to perform a CT scan just to look for signs of kidney cancer. However, in this study because you are already having a CT scan of your lungs, we are offering you the opportunity to add on a CT scan to look for kidney cancer.

For all types of cancer screening, there are benefits but also some downsides. We would like to explain these to you so that you can make up your own mind and decide for yourself if you would like the extra scan.

What are the potential benefits of the extra CT scan?

There is no direct benefit from having the scan itself. However, we believe that there could be some benefit if early, more treatable, cancers are detected. We estimate that in about 5 out of a 1000 people the scan will show evidence of a kidney cancer. These cancers picked up through screening tend to be early and more treatable. However, it has not yet been proven in trials that detecting these cancers by screening reduces deaths – this study is the first step in gathering the evidence to see if this is the case.

The CT scan may also pick-up findings in the kidney which are not cancer but which require further tests or a repeat scan in a few months' time.

Although the extra CT scan is being done to look at the kidneys, it will also include your upper abdomen. This means we may pick up problems in other organs, including the liver, pancreas and aorta. If such problems are detected, you will be referred to the appropriate specialist to diagnose and treat them.

Are there any risks from the extra CT scan?

CT scanners use radiation to produce the pictures of your kidneys. Exposure to radiation can itself cause problems (very rarely actually causing cancer), but by using very modern CT scanners we can reduce the amount of radiation needed. The extra CT scan of the kidneys will slightly increase the amount of radiation you are exposed to. This extra dose is the same amount of radiation that we come into contact within our daily lives over a 12 month period. The likelihood of this scan detecting an early cancer is far greater than the likelihood of the scan causing you harm.

Occasionally, people may have tests or treatments for findings that were not needed. This is because the finding later turns out to be benign (not a cancer) or is a harmless type of kidney cancer (that would not cause problems even if left alone). It is important you are aware of this possibility before having the scan.

For peer review only - http://bmjopen.bmj.com/site/about/guidelines.xhtml

Having the extra CT scan of your kidneys could make you feel anxious or worried about what the scan might show. If a problem is found needing further tests or treatment, this may also cause you worry and anxiety. If you would like to talk to our doctors and nurses they will always be available to discuss any concerns you may have.

What will happen after the scan?
An expert team of doctors will check your CT scan. We will write to you with your results within the next 4 weeks. If you need any other tests or treatment you may need to come to a hospital clinic. Sometimes these tests include extra scans or biopsies. The team of doctors will make sure you are only sent for tests that are necessary. We will take your wishes into account at every step of the process. All decisions about tests or treatment will be made jointly by you and your doctor or nurse.

How accurate is CT screening?
CT scanning is very accurate but not perfect. Very occasionally, the screen will give a normal result, but will fail to pick up an existing cancer. It is very important that you tell your GP if you develop any new symptoms after the CT scan, for example abdominal pain, blood in the urine or unintentional weight loss. If necessary your GP can arrange extra tests.

Is CT screening currently available on the NHS?
CT screening of the kidneys is not yet available on the NHS. We need more evidence before we can decide whether the NHS should offer kidney screening. The Yorkshire Kidney Screening Trial is helping to provide this evidence.

Why have I been invited?
We are inviting all participants in the Yorkshire Lung Screening Trial who attend for their scheduled Lung Health Check follow up to have the additional kidney scan.

Will my information be kept confidential?
Yes. Only the study team at Leeds Teaching Hospitals, the University of Leeds, the University of Cambridge and other healthcare professionals who need access to your medical records will know you are taking part in the study. We will ask if you are happy for us to access your medical records, as well as contact your GP and other doctors about your health both during and for 10 years following the study and to keep this information securely for 15 years. This will allow us to see how any findings identified by the kidney CT scan are managed, and what happens to participants involved in the study over a long period of time. If you agree, we will also keep your GP informed of the results of the additional kidney scan. If you agree to be contacted about potentially taking part in a phone or video interview, then your name and contact details may be passed onto researchers at the University of Cambridge. That information will be stored securely.

If you take part in the questionnaire sub-study, the data you provide will be sent to the University of Cambridge. You will have the choice to either post your completed questionnaire back to the Cambridge research team, or to complete it online, which will then be downloaded by the Cambridge research team. Your questionnaire data will be labelled only with your YKST study ID and will not include your name or address or be labelled with anything else identifiable.

Paper questionnaires will be sent to a professional data entry company who will sign a confidentiality agreement so that your data cannot be shared. They will enter the questionnaire data electronically and return the paper copies back to the University of Cambridge. The paper copies will be kept in locked filing cabinets until the data from them have been checked for accuracy. They will then be destroyed.

We plan to share study information with other researchers, including those at the University of Cambridge, but we will not share any data that identifies you. We will publish the results of the study in medical

For peer review only - http://bmjopen.bmj.com/site/about/guidelines.xhtml

journals, but again you will not be identified. If you agree we will keep your CT scans and other linked information about you for use in future research projects. These projects might not involve the original research team and could be either an academic or commercial partner. We will make sure that anyone we share data with has permission for their research and we will use the minimum data necessary.

The University of Leeds is the sponsor for the study which means they are responsible for the research. The University and Leeds Teaching Hospitals' are joint data controller for the study. This means they will look after your data and make sure that it is used properly. This will include checking that the information collected about you is accurate. Only the people who carry out the checks will see information about you. The information stored in the University will include your NHS number and Date of Birth, but will not include your name, address or telephone number. No one from the University will contact you as part of this checking process.

You can stop being part of the study at any time, without giving a reason. We will then stop collecting new data about you but we will keep information about you that we already have.

You can find out more about how we use your information by contacting the research team using the contact details at the end of this form or the University of Leeds data protection officer at: dpo@leeds.ac.uk

More information can also be found by visiting:
www.hra.nhs.uk/information-about-patients/
https://dataprotection.leeds.ac.uk/research-participant-privacy-notice/
http://www.leedsth.nhs.uk/patients-visitors/patient-and-visitor-information/how-weuse-your-data/

What will happen to the results of the research study?

- At the end of the study a summary of our results will be published on the study website which can be accessed at www.ykst.org.uk.
- The results of this research will inform recommendations about how the NHS delivers cancer screening tests.
- The results will be reported in scientific journals and presented at academic conferences. While we may use your quotes, you will never be identified.

Who do I contact if I have a problem?
If you have any questions or concerns about any element of the study, please contact the Yorkshire Kidney Screening Trial team on 07708673022. Calls will be answered during office hours, alternatively you can leave a message and one of the team will get back to you.
If you are unhappy with the care you received as part of the study, you can contact the NHS Patient Advice and Liaison Service (PALS) on 0113 206 6261. It is very unlikely that anything will go wrong. The University of Leeds has insurance to compensate you if you should come to any harm.

Further information and contact details

Chief Investigator:
Professor Grant Stewart, Professor of Surgical Oncology, Department of Surgery, University of Cambridge
Email: yorkshirekidneyscreen@nhs.net

Research Nurse: Fiona Farquhar
Telephone no: 0113 206 0473

For peer review only - http://bmjopen.bmj.com/site/about/guidelines.xhtml

Thank you for taking the time to read this information sheet and for thinking about taking part. Taking part in this trial is purely voluntary and the additional kidney scan will not happen unless you have signed the accompanying Patient Consent Form.

For peer review only

For peer review only - http://bmjopen.bmj.com/site/about/guidelines.xhtml
